# Integrated Cells and Collagen Fibers Spatial Image Analysis

**DOI:** 10.3389/fbinf.2021.758775

**Published:** 2021-11-08

**Authors:** Georgii Vasiukov, Tatiana Novitskaya, Maria-Fernanda Senosain, Alex Camai, Anna Menshikh, Pierre Massion, Andries Zijlstra, Sergey Novitskiy

**Affiliations:** 1 Department of Medicine, Division of Allergy, Pulmonary, Critical Care Medicine, Vanderbilt, University Medical Center, Nashville, TN, United States,; 2 Department of Pathology, Microbiology, And Immunology, Vanderbilt University Medical Center, Nashville, TN, United States,; 3 Department of Medicine, Division of Nephrology, Vanderbilt University Medical Center, Nashville, TN, United States

**Keywords:** image analysis, ECM–extracellular matrix, spatial analysis, fibers, image processing, collagen fiber (CF)

## Abstract

Modern technologies designed for tissue structure visualization like brightfield microscopy, fluorescent microscopy, mass cytometry imaging (MCI) and mass spectrometry imaging (MSI) provide large amounts of quantitative and spatial information about cells and tissue structures like vessels, bronchioles etc. Many published reports have demonstrated that the structural features of cells and extracellular matrix (ECM) and their interactions strongly predict disease development and progression. Computational image analysis methods in combination with spatial analysis and machine learning can reveal novel structural patterns in normal and diseased tissue. Here, we have developed a Python package designed for integrated analysis of cells and ECM in a spatially dependent manner. The package performs segmentation, labeling and feature analysis of ECM fibers, combines this information with pre-generated single-cell based datasets and realizes cell-cell and cell-fiber spatial analysis. To demonstrate performance and compatibility of our computational tool, we integrated it with a pipeline designed for cell segmentation, classification, and feature analysis in the KNIME analytical platform. For validation, we used a set of mouse mammary gland tumors and human lung adenocarcinoma tissue samples stained for multiple cellular markers and collagen as the main ECM protein. The developed package provides sufficient performance and precision to be used as a novel method to investigate cell-ECM relationships in the tissue, as well as detect structural patterns correlated with specific disease outcomes.

## INTRODUCTION

Imaging is the most appropriate method for tissue structure analysis, because it can be implemented without or with minimal integrity disruption. Imaging techniques can be performed without invasive procedures (CT, MRI) or harvesting biological material (histology-based techniques). Modern histopathological methods can detect and visualize a broad variety of markers despite their chemical nature. This fact makes it possible to establish a bridge between morphology and molecular pathology of the tissue and, in combination with powerful computational methods, detect novel structural patterns related with disease outcomes (Roeder et al., 2012; Schapiro et al., 2017; Czech et al., 2019). Modern research and clinical imaging systems are accurate, fast, and able to store large amounts of data. For example, fluorescent whole-slide scanners generate full-scale images of histological slides, which then can be saved and analyzed later by different image analysis software (Himmel et al., 2018). This makes developing efficient, flexible and versatile computational image analysis approaches a very important mission.

Tissue is a complex system formed by cellular and non-cellular components. Cellular components include diverse groups of cells such as epitheliocytes, immune cells, fibroblasts, endotheliocytes, and adipocytes, among others. Non-cellular components are formed by ECM, web-like integrated structures which consist of fibrillar and non-fibrillar proteins (Pickup et al., 2013; Bhat and Bissell, 2014; Cho et al., 2015). The fibrillar part of the ECM is formed by various groups of proteins like collagen, fibronectin, elastin and laminin. Non-fibrillar ECM consists mostly of proteoglycans (Järveläinen et al., 2009). ECM composition and topology are unique for each tissue and generated during tissue development by dynamic and reciprocal interactions between cells and their microenvironment (Frantz et al., 2010). The structures of ECM perform a broad variety of biological functions like reception and transmission of mechanical signals to cells, storing growth factors and other cytokines, and presenting ligands to their receptors on cells (Leitinger and Hohenester, 2007). However, the most important role of ECM is providing an adhesive substrate and scaffold for other parts of tissue. Deep integration of ECM with all tissue components causes dynamic changes in its structure during normal and pathological states. ECM structures, especially the fibrous components, can be characterized by geometrical (length, width, straightness), spatial (alignment) and physical (stiffness and elasticity) properties (Provenzano et al., 2009; Conklin et al., 2011; Hanley et al., 2016). Collagen is the most abundant protein in ECM and determines most physical and geometrical properties of ECM in tissue. Many studies have shown that geometrical, physical and spatial features of collagen fibers are correlated with cancer outcomes (Provenzano et al., 2009; Frantz et al., 2010; Conklin et al., 2011; Wen et al., 2014; Hanley et al., 2016). Changes in ECM properties, in turn, affect the behavior of cells and their composition in tissue. For instance, in stiffer tumor microenvironments, epithelial cells and fibroblasts are characterized by increased contractility. Increased stiffness and collagen deposition affect aggressive behavior of tumor cells and infiltration of tumor nests by immune cells (Paszek and Weaver, 2004; Paszek et al., 2005; Butcher et al., 2009; Erler and Weaver, 2009; Frantz et al., 2010). On the other hand, investigations of the tissue structure during different pathological states often gives controversial results. In our opinion, for better understanding of the mechanisms that take place in tissue during different pathological processes, combined analysis of cellular and non-cellular tissue components is required to improve depth and quality of approaches that already exist.

The complexity of tissue structure requires novel methods of investigation that will give more comprehensive information, including morphology and spatial distribution of different tissue objects. Computational image processing usually proceeds through the following steps: image acquisition, preprocessing, segmentation, morphological image processing, spatial analysis (neighborhood analysis), postprocessing, visualization and validation (Roeder et al., 2012; Jost and Waters, 2019). Tissue spatial analysis is a group of methods aimed at studying spatial relationships between various types of cells and their microenvironment. These methods attempt to identify global features and structural patterns in normal and pathological tissues. Computational methods include calculating distances between objects (cells, ECM fibers etc.), implementing nearest neighbor analysis, clustering, and graph-based analysis, combining different statistical and machine learning methods (Roeder et al., 2012; Heindl et al., 2015; Parra, 2021). Existing open-source and commercial software is designed mostly for separate analyses of cells, fibers, blood vessels or other structures in the tissue. Novel methods mostly aim to improve and increase the quality of current image processing approaches like denoising, object segmentation and labeling, classification, and 2D and 3D visualization. For integrated analysis of cellular and non-cellular components of tissue structure, computational methods must be able to perform image processing on both simultaneously. The approach to image analysis in cells and fibers is different. For instance, methods for separating background from foreground and single-cell segmentation and labeling demonstrate low efficacy regarding fiber analysis because of differences in geometrical characteristics of objects (Wu et al., 2003; Stein et al., 2008; Bayan et al., 2009; Rubbens et al., 2009; Pijanka et al., 2019).

Here, we have developed a Python package for integrated spatial analysis of cellular and fibrous components of tissue. Our algorithm performs segmentation of ECM fibers, assess their morphology and spatial distribution and combines this information with datasets that include information about cellular localization, geometrical features, and phenotype, generated by other computational approaches. We implemented our algorithm within the KNIME analytical platform environment (Dietz and Berthold, 2016). This computational tool demonstrates a broad variety of methods that can be used for a tremendous number of tasks like mathematics, chemistry, data analysis, and bioinformatics. In addition, KNIME supports implementation of different programming languages like MATLAB (http://www.mathworks.com/products/matlab/), R (http://www.r-project. org/), or Python (https://www.python.org/) within its environment (Berg et al., 2019; Vasiukov et al., 2020; Senosain et al., 2021). Using KNIME, we combined our algorithm with a computational method we used in our previous publication which was designed for cell segmentation, labeling and classification (Vasiukov et al., 2020; Senosain et al., 2021). Algorithm validation confirms that by combining integrated analysis of cellular and acellular components of tissue, it is possible to reveal novel structural patterns that can be used to observe disease pathogenesis.

## MATERIALS AND METHODS

### Sample Description

Mouse mammary gland tumor tissue samples were harvested from PyMT (WT) and PyMT/TGFβRII^LysM^ (KO) mice as described in our previous work where we found increased ECM deposition in PyMT/TGFβRII^LysM^ samples (Stringer et al., 2021). Human lung adenocarcinoma tissue samples were collected from 14 patients after surgery under IRB approved protocol 000616 at Vanderbilt University Medical Center. Using Computer-Aided Nodule Assessment and Risk Yield (CANARY) software we stratified the patients into two risk groups (Indolent and Aggressive) regarding behavior of adenocarcinoma as we showed in (Senosain et al., 2021). Two TMA cores from each tissue sample were processed.

### Tissue Preparation and Staining

Tissues were fixed in 10% neutral buffered formalin for 24 h at room temperature. Fixed samples were embedded in paraffine blocks, and 5 μm sections were cut. Tissue microarrays (TMA) were generated from patient’s material. All cores were examined by a pathologist to exclude tissue with massive necrosis areas, large blood vessels and artifacts. H&E staining was used to evaluate core quality. For visualization of collagen in the tissue samples we used picrosirius red staining and CHP–Collagen Hybridizing Peptide (3Helix, United States) staining labeled with Cy3 dye. Multiplex immunofluorescent (IF) staining was used to combine CHP staining with a set of cellular markers (Senosain et al., 2021). Epithelial/tumor cells were visualized with anti-PanCK-Alexa Fluor 488 antibodies (eBioscience, United States), immune cells with - anti-CD45 antibodies (Biolegend, United States) + Fab anti-mouse-Cy3 antibodies (Jackson ImmunoResearch, United States), and T-cells with anti-CD3 antibodies (Agilent Dako, United States) + anti-rabbit-Cy5 antibodies (Thermo Fisher Scientific, United States). Nuclei were visualized by Gold Antifade Mountant with DAPI (Thermo Fisher Scientific, United States). Images from Picrosirius red stained samples were obtained using the Keyence BZ-X710 microscope (KEYENCE, United States) using ×20 objective and Texas Red filter (excitation wavelength 560/40 nm, emission wavelength 630/75 nm). Whole image scanning was performed on the Apiro Versa 200 (Leica, United States) with Texas Red filter (excitation wavelength 560/55 nm, emission wavelength 645/75 nm), Spectrum Green filter (excitation wavelength 480/30 nm, emission wavelength 535/40 nm), Cy5/AlexaFluor 647 filter (excitation wavelength 620/50 nm, emission wavelength 690/50 nm), Cy7 filter (excitation wavelength 720/75 nm, emission wavelength 810/90 nm) and DAPI filter (excitation wavelength 350/50 nm, emission wavelength 460/50 nm).

### Software and Image Analysis

The KNIME (KNIME 4.1.2) analytical platform was used as an environment for integration of our Python package for fiber segmentation and spatial analysis with workflow for single-cell analysis of multiplexed fluorescent stained images. Segmentation, labeling and classification of cells was performed as described in our previous work (Senosain et al., 2021). Quantitative information from single-cell data (such as X, Y coordinates etc.) was used for spatial analysis using developed Python package (Python 3.7). Python nodes were incorporated in this pipeline for collagen fiber segmentation, labeling and analysis of geometrical features and spatial analysis of cells and fibers.

### Performance Tests and Validation

Set of mouse and human cancer tissue samples was used to implement performance tests and validation of the algorithm. Breast cancer image size: 1920 × 1440 pixels, human lung adenocarcinoma (whole slide scans) image size: 5120 × 5120 pixels. Collagen fibers were manually annotated by pathologist and their length was measured using ImageJ. These results were used as a ground truth for further tests. Number of highlighted fibers and their length were chosen as only features for ground truth due to restrictions of manual measurements of other parameters (width, angle etc.). Mean value of the parameters was calculated for each image. The results were presented as average for the set of images. Accuracy test was performed as a comparison to ground truth and to results obtained using CT-FIRE algorithm (Bredfeldt et al., 2014a). Performance time test was realized using a set of 50 images. The results were compared to the CT-FIRE algorithm (Bredfeldt et al., 2014a). Neighborhood outline for breast cancer samples was set as 100 pixels and for lung cancer tissue as 250 pixels and was equal to ~50 μm and ~100 μm in diameter respectively.

### Computer System’s Specifications

DELL Precision T7920, two Intel Xeon Gold 6136 Processors (24 Core, 3.0 Ghz, 24.75 M Cache each), 128 Gb RAM.

### Availability and Usage

The Microsa source code, including trial dataset and user manual, is available at https://github.com/VGeorgii/Microsa.

### Statistical Analysis

Results were presented as mean ± SEM. Two-group comparison was performed using two-sample t tests or Wilcoxon Rank-Sum test as appropriate. P values less than 0.05 were considered statistically significant. Hierarchical cluster analysis was performed using Ward’s method for Euclidian distances.

## RESULTS

### Significance and Package Design

**FIGURE 1 |:**
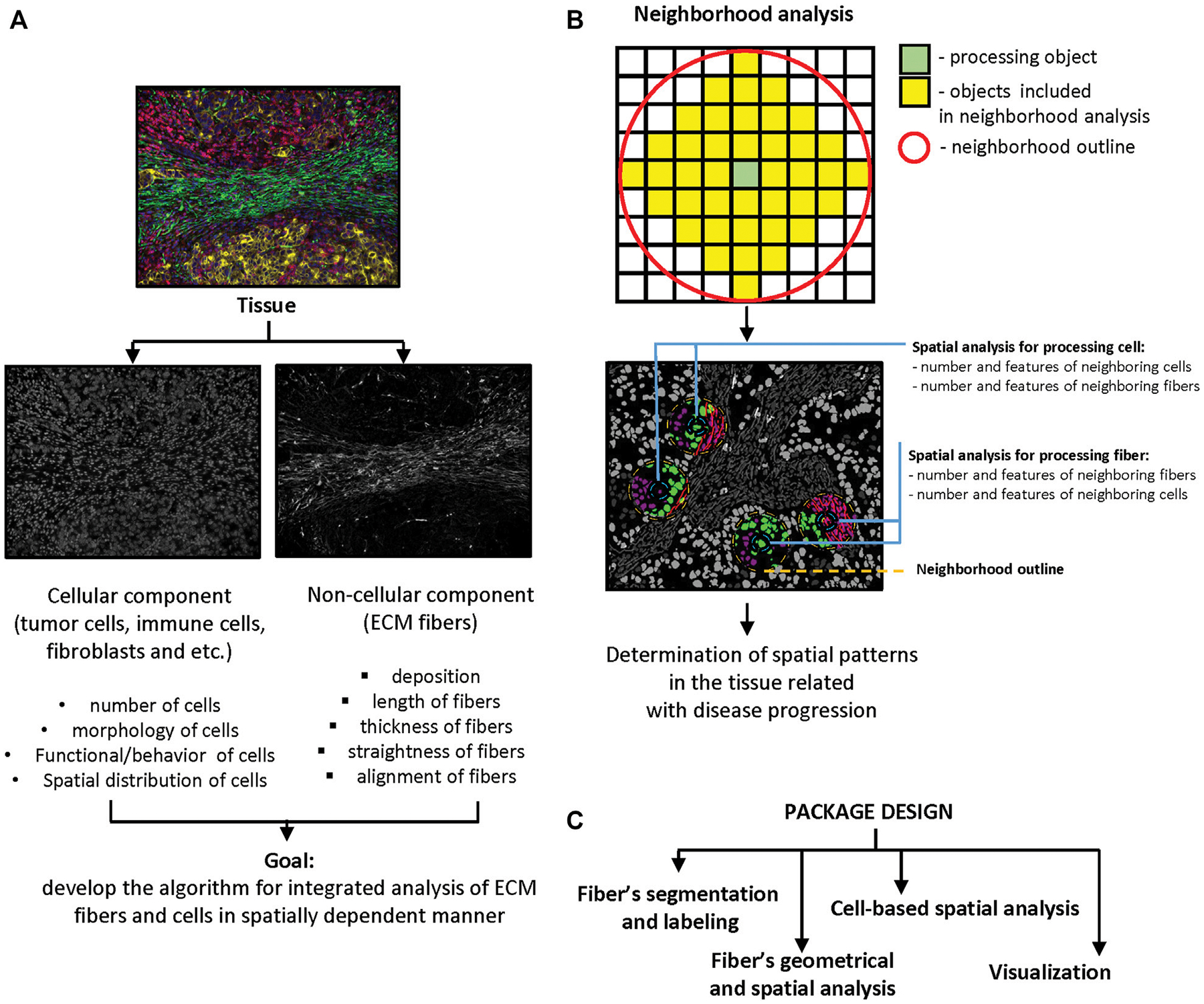
Significance and package design. **(A)** Main goal of package design. The scheme demonstrates the idea of combined analysis of cellular and non-cellular components of tumor microenvironment in spatially dependent manner. **(B)** Principle of neighborhood analysis. Processing object (cell or fiber) and radius of the neighborhood outline need to be defined first. The features of objects that are localized in the outline analyzed as properties of processing object. The number of neighboring objects and their features need to be calculated for every processing object in the tissue. **(C)** Package design. Package contains four modules: fibers segmentation, fibers geometrical and spatial analysis, cell-based spatial analysis, and visualization.

Implementation of computational image analysis for scientific and clinical purposes is a robust, effective, and precise approach. The complexity of tissue structure requires novel methods and technologies for image acquisition and processing that will extend the dimensionality of extracted information. Tissues are characterized by a combination of objects with certain morphology, number, and spatial distribution. Cells in tissue can interact by close (cell junctions) and distant (cytokines, chemokines, metabolites etc.) types of communication. The distance between objects plays a crucial role in the determining the effects of such interactions. Our goal was to develop and test computational methods which would be able to combine analysis of cellular and fibrous constituents of tissue structure in a spatially dependent manner (Figure 1A). Spatial analysis can be implemented to identify of structural patterns in tissue that help to decipher disease pathogenesis or that can be used for outcome prediction. There are several approaches that can be used for spatial analysis: distance calculation (Parra, 2021), spatial homogeneity assessment (Holmes et al., 2009), and clustering or graph algorithms (Roeder et al., 2012; Heindl et al., 2015; Parra, 2021). Our tool implements distance calculation. This approach can be realized in two ways. The first approach (“number”) is based on the analysis of neighborhood outlines around processed cells or other objects in the tissue. The radius of outline is set by the user and determined by the type of interactions the researcher is interested in (Figure 1B). A small radius allows the researchers to calculate objects in close contact to the processing object, while a bigger radius can be used to calculate the number of objects in distant contact. The second approach (“distance”) involves calculating the distance between the processed object and other objects of interest. For instance, the distance between a tumor cell and the closest T-cell, or between a T cell and a blood vessel etc. After that, extracted information can be passed to K-nearest neighbors, N-distance, or self-organizing map algorithms for identification of neighborhoods with similar features (Roeder et al., 2012; Heindl et al., 2015; Parra, 2021).

To perform segmentation, labeling and geometrical feature analysis of fibers we used the Python programming language. This decision was determined by the flexibility of Python and diversity of packages designed for image processing that demonstrate good performance and efficacy. The developed Python package was designed for assessment of fiber features and combination of extracted information with cell-based datasets for spatial analysis. Our package uses several popular python libraries: Pandas (https://pandas.pydata.org/), SciPy (Virtanen et al., 2020), NumPy (Harris et al., 2020) and Scikit-image (van der Walt et al., 2014), Matplotlib (Hunter, 2007). The package consists of four modules: i) fiber segmentation and labeling, ii) fiber geometrical feature calculation and spatial analysis, iii) cell-based spatial analysis, and iv) visualization (Figure 1C).

### Fiber Segmentation, Labeling and Feature Analysis

Before implementing of these modules of our Python package, the user needs to perform foreground-background separation. By our experience, the most efficient methods are simple thresholding and Frangi filter-based thresholding (Frangi et al., 1998; Hemler et al., 2004; Shi and Yang, 2009; Park et al., 2013; Comin et al., 2014). Simple thresholding efficiently processes images with small numbers of fibers and low levels of background. The Frangi filter method was designed specifically for objects that form net-like structures (blood vessels, fibers etc), which can better identify densely packed fibers (Frangi et al., 1998).

**FIGURE 2 |:**
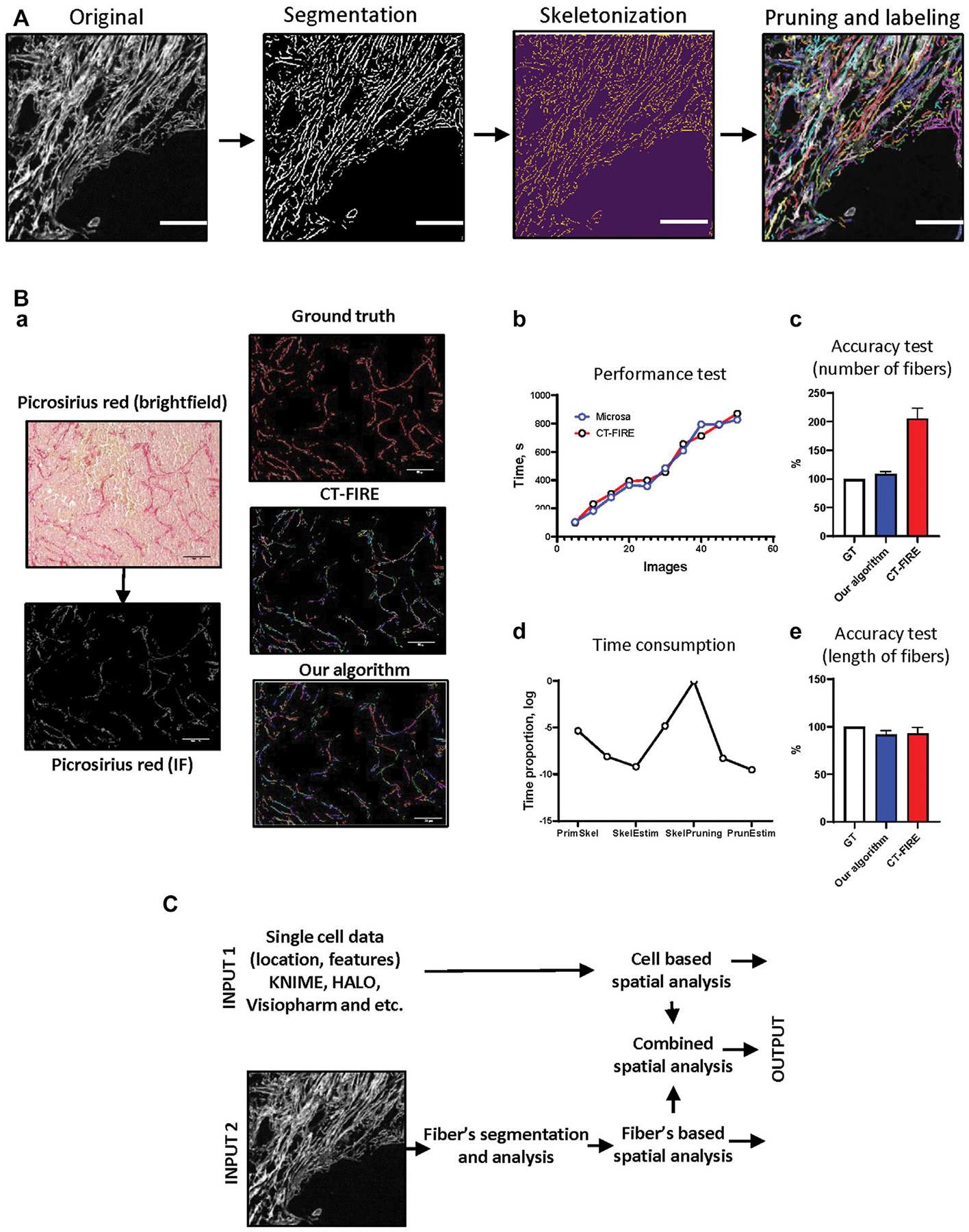
Package implication. **(A)** The algorithm for fiber segmentation and labeling. “Original image”—image with visualized fibers. “Segmentation”—background/foreground separation, highlighting of separated fibers using “Original image”, “Skeletonization”—erosion of single fibers until the thickness is equal to 1 pixel, “Pruning”–removing additional brunches, “Labeling”—indexing of segmented single fibers. Scale bar 100 μm. **(B)** Algorithm execution tests. Mouse mammary gland tumor tissue was analyzed manually (ground truth), using CT-FIRE software and our algorithm (Microsa) **(a)**. Collagen fibers were visualized using Picrosirius red staining. Image size–1920 × 1440 pixels, scale bar—100 μm. Performance test of CT-FIRE and our algorithm demonstrates time required for the analysis of different numbers of images **(b)**. Time consumption test shows proportion of time required for each procedure during fiber segmentation and labeling algorithm execution **(d)**. Accuracy tests demonstrate precision of CT-FIRE and our algorithm performance in comparison to manually analyzed data **(c, e)**. **(C)** The scheme of cell and fiber segmentation and labeling algorithms with subsequent spatial analysis. Three types of output: cell-based spatial analysis, fibers-based spatial analysis and combined spatial analysis.

ECM fibers in the tissue often have complex curve-like structure with different lengths, widths, and linearities, and often have additional branches. To perform single-fiber analysis, we decided to process each fiber as a curve with no branches, which reflects general geometrical features and does not have intersections with another fibers. We found that the most efficient way to generate a simplified model of the fibers is to use thinning or skeletonization algorithms (Comin et al., 2014). However, because of the complexity of collagen fiber shapes the algorithm we used for skeletonization generated multiple artifacts that appeared as spikes or additional small branches. We therefore implemented an extra process, which helps to remove these artifacts from the fiber skeleton. The visualization module can be used to overlay segmented fibers with the original image to control quality of segmentation and labeling (Figure 2A). After fiber segmentation and labeling is complete, the geometrical feature calculation module can be used to extract fiber properties.

### Length

This parameter represents the length of the longest branch of the fiber. The value is calculated as length of the curve between two endpoints.

### Thickness

This parameter represents average distance from pixels of the fiber’s skeleton to the outline of fiber’s mask.

$$\text{Thickness}=\left(\sum_{i=0}^{N}di\right)/N$$

where d–distance from pixel to fiber’s mask outline, N number of the pixel in the fiber’s skeleton.

### Angle

This parameter represents the angle between the X axis and a straight line between the fiber’s curve endpoints. Angle can take values between 0 and 180°.

### Intensity

This parameter represents average intensity of the fiber’s skeleton pixels

$$\text{Intensity}=\left(\sum_{i=0}^{N}Ii\right)/N$$

where I–pixel intensity (from 0 to 255), N–total number of pixels in the fiber’s skeleton.

### Straightness

This coefficient represents how close the shape of the fiber is to straight line. It takes the values from 0 to 1.

Straightness=l/L

where l–length of fiber’s curve, L–Euclidian distance between endpoints.

### Alignment

This coefficient reflects the alignment of processing fiber and its neighboring fibers. It can take values from 0 (not aligned) to 1 (absolutely aligned). The number of neighboring fibers depends on radius of the neighborhood outline which can be regulated.

$$\text{Alignment}=\left(\sum_{i=0}^{n}\text{cos}\left(\alpha i\right)\right)/n$$

where *α* – angle between processing fiber and neighboring fiber *i*, n–total number of neighboring fibers.

The output of these modules is a dataset which contains information about each fiber’s location (XY coordinates) and geometrical features. Implementation of cell-based spatial analysis gives an opportunity to investigate features of fibers co-localized with processing cells.

To test the performance of fiber segmentation and fiber features analysis algorithms we used a set of images obtained from mouse mammary gland tumor samples stained with Picrosirius red (Figures 2Ba). The results of our algorithm have been compared to other software designed for fiber analysis–CT-FIRE (Stein et al., 2008; Bredfeldt et al., 2014a). This approach was implemented in multiple works (Bredfeldt et al., 2014a; Bredfeldt et al., 2014b). Our team was inspired by idea that is realized in CT-FIRE–to track and analyze geometrical features of each fiber in the tissue image. CT-FIRE uses different method for segmentation and labeling of fibers and we were interested to compare performance of our method and CT-FIRE. Both algorithms were executed using parallel computation mode (13 cores). We found that our approach demonstrated better results (Figure 2Bb). To assess the accuracy of our developed algorithm we used a set of samples manually annotated by pathologist and compared with results generated by our approach and CT-FIRE. We found that number of fibers segmented and labeled by our method is closer to the ground truth (Figure 2Bc)). Both approaches demonstrated good accuracy estimating length of segmented fibers (Figure 2Be). Additional analysis demonstrated that pruning is the most time-consuming step in the fiber segmentation algorithm and needs further amendments or revision (Figure 2Bd).

### Combination With Cell Related Data

This package was designed to combine single-cell and single-fiber datasets in spatially dependent manner. The generated data can be used for statistics and machine learning methods. Cell segmentation and labeling must be performed using appropriate software or algorithms (QuPath (Bankhead et al., 2017), KNIME (Dietz and Berthold, 2016)) and generated single-cell datasets can be utilized as an input for spatial analysis algorithm. To generate single-fiber datasets the user can use images with fibers visualized by brightfield staining (Picrosirius red, Trichrome blue, IHC) or IF staining (antibodies against collagen CHP/3Helix etc.) techniques and apply modules for fiber segmentation and fiber geometrical feature analysis from our package, or utilize other methods or software designed for that purpose. Using modules for spatial analysis, the user can generate additional information for each labeled cell or fiber based on objects (cells or fibers) localized in a neighborhood outline of a given radius (Figure 2C).

### Validation

To validate the accuracy and performance of our developed package and test its integration with other algorithms and software, we used several sets of tissue section slides stained by different techniques. Geometrical features, deposition of collagen fibers and their visualization methods can significantly affect algorithm performance. In our current work, we used Picrosirius red and collagen hybridizing peptide (CHP/3Helix) staining to visualize fibers.

**FIGURE 3 |:**
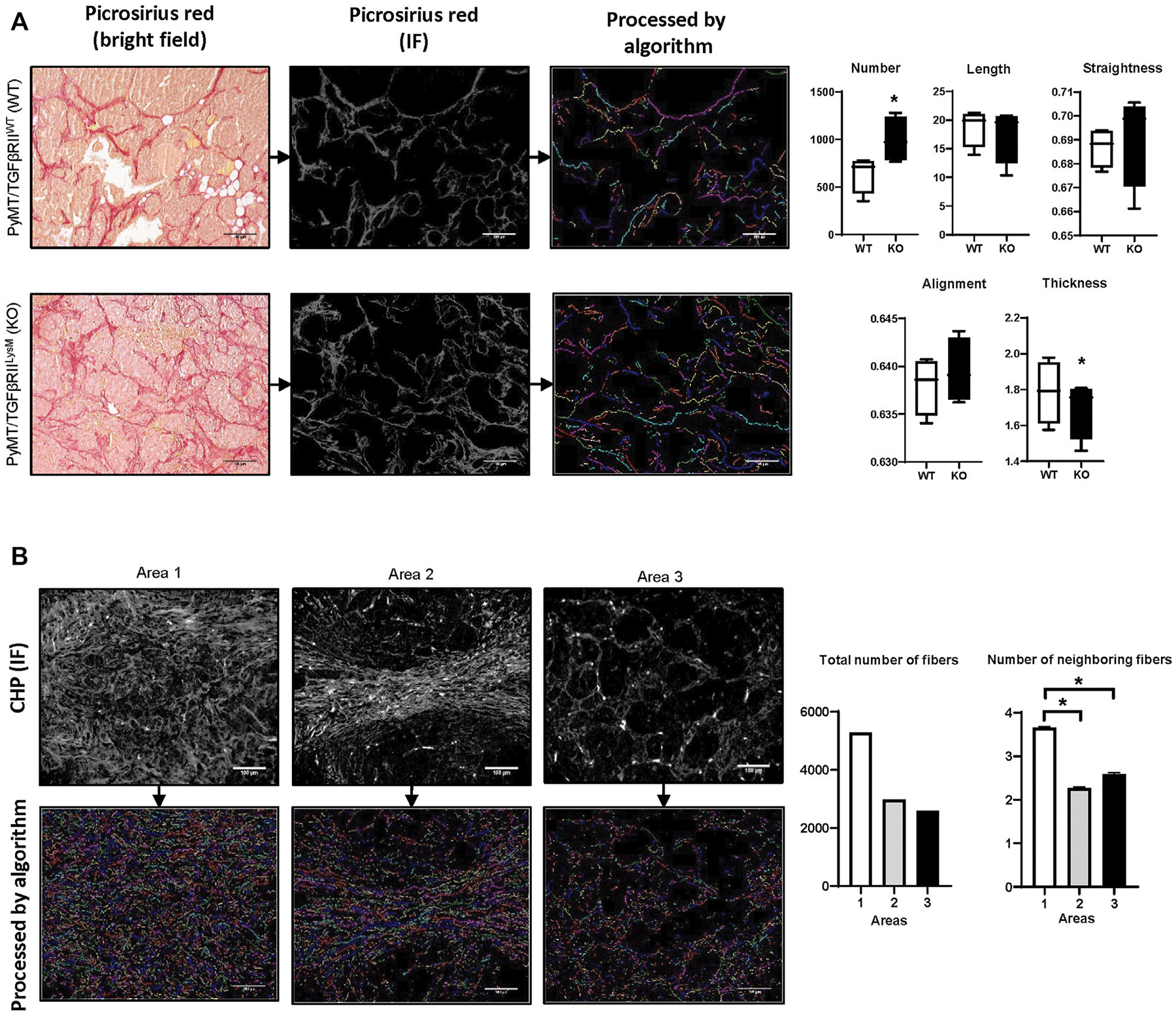
Examples of algorithm usage. **(A)** Implementation of module for fiber geometrical analysis of PyMT mouse mammary tumor tissue from WT and TGFβRII KO groups (Vasiukov et al., 2020). Collagen fibers were visualized using Picrosirius red staining. Length, width, straightness, and alignment were calculated for each fiber and presented as average. *−*p* < 0,05. Image size–1920 × 1440, scale bar—100 μm. **(B)** Implementation of modules for fiber labeling and spatial analysis for human breast cancer tissue stained by CHP-Cy3 (3Helix, United States). Number of neighboring fibers was calculated as total number of fibers within neighborhood outline for each fiber. Radius of the fiber’s neighborhood outline—~50 μm. Image size–1920 × 1440, scale bar—100 μm.

In our previous work we have used the PyMT mouse model (spontaneous mammary gland tumor formation) (Vasiukov et al., 2020). This work demonstrated that lack of TGFβ signaling in myeloid cells (PyMT/TGFβRII^LysM^) is related to reduced tumor growth and increased collagen deposition. Here, for visualization of collagen fibers we have used Picrosirius red staining with subsequent calculation of collagen deposition. One of the advantages of Picrosirius red staining is the ability to use polarized light to identify immature and mature collagen fibers. In our work we demonstrated that in the KO group tumor tissue had higher amounts of immature collagen that indicate more intensive ECM remodeling. Using the fiber segmentation module in our developed package, we detected increased number of collagen fibers in tumor tissue in the PyMT /TGFβRII^LysM^ group. This data confirms results we obtained in our previous work using different calculation methods. In addition, analysis of geometrical features of collagen fibers demonstrated tendency that collagen fibers in the PyMT /TGFβRII^LysM^ group have increased straightness and alignment, but decreased thickness in comparison to PyMT/TGFβRII^WT^ group (Figure 3A).

For collagen detection in human breast cancer tissue samples, we used CHP staining. CHP visualizes collagen well with low background noise. Moreover, this method can be used for multiplex IF staining that allows simultaneous detection of different biological markers. We visually chose three different areas that were characterized by different collagen deposition and fiber spatial distribution. Analysis demonstrated that Area 1 had the highest number of collagen fibers, whereas Area three had the lowest number. Investigation of fibers co-localization demonstrated similar results. Area 1 showed the highest rate of fiber co-localization (Figure 3B).

**FIGURE 4 |:**
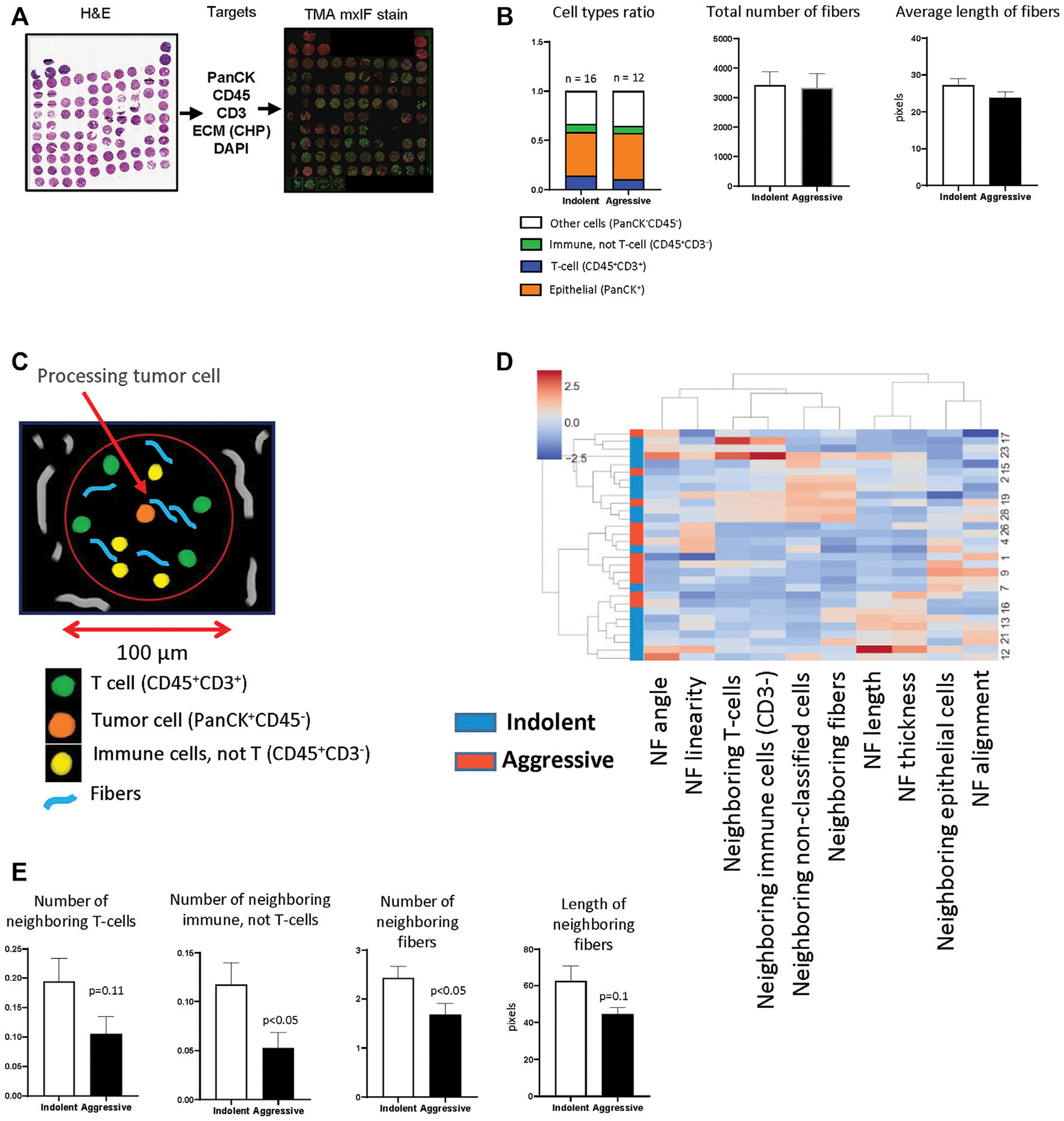
TMA analysis. **(A)** TMA was generated from lung tissue blocks from patients with lung adenocarcinoma as described. Two tissue cores were used to represent one patient. Fluorescent staining was performed for PanCK, CD45, CD3, DAPI. **(B)** The analysis of basic features of tumor tissue. **(C)** Diagram demonstrates principle of implemented spatial analysis. Tumor cells were used as processing objects, neighborhood outline was equal to 100 μm, all cells and fibers within the neighborhood outline were counted and analyzed. **(D)** Hierarchical cluster analysis based on spatial features of tumor cells (NF–neighboring fibers). **(E)** Comparison of spatial features of tumor cells in indolent and aggressive groups.

As was mentioned above, CHP staining is suitable for multiplex IF that allows simultaneous visualization of cellular markers and collagen fibers and can be used for integrated analysis to reveal relationships between them. In our previous work (Senosain et al., 2021), we used a panel of antibodies for visualizing of epithelial/tumor cells (PanCK), immune cells (CD45) and T-cells (CD3) (Figure 4A) to reveal relationships between anticancer immune response and behavior (indolent or aggressive) of human lung adenocarcinomas. To perform cell’s segmentation, labeling and classification we utilized workflow developed in KNIME. In current work, we additionally performed CHP (3Helix) staining to identify collagen fibers. To analyze tumor tissue from patients with indolent and aggressive lung adenocarcinoma, we used dataset generated in our previous work (Senosain et al., 2021). First, we performed analysis of basic parameters of tumor tissue (total number of different type of cells, total number of collagen fibers and their morphology). We did not find a significant difference in cellular composition between indolent and aggressive groups of patients. Calculation of collagen fibers showed that there is no difference in number of fibers and their geometrical features (Figure 4B). Next, we performed spatial analysis and used tumor cell as a processing object. Neighborhood outline was equal to 100 μm. Cells and collagen fibers within the neighborhood outline was counted and geometrical features of fibers were estimated (Figure 4C). The analysis revealed that tumor cells from the aggressive group were co-localized with lower number of CD3^−^ immune cells. In addition, tumor cells from aggressive lung adenocarcinomas were co-localized with lower number of collagen fibers and these fibers generally had smaller length (Figures 4D,E).

## DISCUSSION

Rapid development of technologies for biological image acquisition and data storage presents an opportunity for investigators to discover new mechanisms related to different diseases development. Engineering novel computational approaches for image analysis allows investigators to obtain detailed quantitative information from large amounts of data. Studying complex, integrated systems like tissue needs further development with new computational methods able to elucidate properties of tissue components individually and as a part of a structural consortium. (Reis-Sobreiro et al., 2018), (Senosain et al., 2021).

Phenotype and functions of cells in the tissue are highly determined by their microenvironment, which is formed by co-localized cells and ECM structures. Multiparametric image processing reveals structural patterns of tissue that have not been elucidated previously (Schapiro et al., 2017). The topography of tissue in normal and pathological states can be deciphered by implementation of spatial analysis. In this study, for the first time, we have developed an approach which allows researchers to combine the analysis of tissue on a single-cell level with assessment of ECM structure in spatially dependent manner. Capturing structural patterns determined by spatial analysis can be achieved through several approaches: distance calculation, spatial homogeneity assessment, and implementation of clustering or graph algorithms. For instance, Balsat et al. measured the distance between lymphatic vessels and the epithelial/tumor edges and demonstrated that the transformation zone of the benign cervix promotes lymphangiogenic process (Balsat et al., 2014). Feichtenbeiner et al. performed cell-to-cell distance calculation and showed the importance of FoxP3+ and CD8^+^ T-cell co-localization for gastric cancer patient outcomes (Feichtenbeiner et al., 2014). Spatial homogeneity in a set of objects can be estimated by K- and L-functions. This approach was used by Holmes et al. for analysis of spatial data on T-cells and B-cells (Holmes et al., 2009). Clustering methods such as density-based clustering (Chen et al., 2002) or K-means clustering algorithm can define a number of subgroups (clusters) in dataset. This is possible using HistoCAT software (Schapiro et al., 2017), MATLAB, R, or Python programming languages. Because we investigated spatial relationships between objects with different structural natures (cells and fibers) we decided to use an approach for spatial analysis based on measurement of distances between objects with subsequent assessment of those that localized in a defined neighborhood outline.

Integration of cell and fiber analyses requires generation of two types of datasets: single-cell and single-fiber. Datasets that contain single-cell information must be generated by software and computational methods designed for this task. The most popular open source tools that can be used: ImageJ (Rueden et al., 2017), FIJI, HistoCAT (Schapiro et al., 2017), CODEX toolkit (Goltsev et al., 2018), KNIME (Dietz and Berthold, 2016), Ilastik (Berg et al., 2019), CellProfiler (Carpenter et al., 2006) and Cytokit (Czech et al., 2019). Another option is to use programming languages such as Python, R and MATLAB that have powerful image analysis packages. Generated single-cell datasets can be used as an input for spatial analysis module for neighborhood assessment of labeled cells. We used KNIME because of its high flexibility and performance. The developed pipeline gave us an opportunity to perform segmentation and labeling of cells and fibers and spatial analysis in a fully automated manner.

Annotation of fiber geometrical and spatial features is a nontrivial task. Computational methods designed for cell segmentation and analysis do not demonstrate good performance for fibers due to the high complexity of fiber geometry and deposition. There are several approaches that can be used for fiber analysis: Hessian matrix (Rubbens et al., 2009), Fourier (Pijanka et al., 2019), and Hough transform (Bayan et al., 2009), curvelet transform (Bredfeldt et al., 2014a), directional filtering (Wen et al., 2014), and fiber tracking (Wu et al., 2003; Stein et al., 2008; Bredfeldt et al., 2014a). Integrated analysis of cellular and fibrillar components of the tissue requires appropriate methods for visualization of this structures. In this research work we used CHP staining to indicate collagen fibers and combination of cellular markers to detect and classify different types of cells. However, implementation of second harmonic generation (SHG) imaging combined with any staining techniques is appropriate method for integrated analysis of cells and ECM. SHG does not need labeling of collagen fibers and can be realized on samples stained by H&E or other methods. To integrate single-cell and single fiber-analysis in a spatially dependent manner, a labeling procedure must be implemented which assigns an index to each segmented fiber and cell. After labeling, additional methods can be used to calculate object localization (coordinates), morphology and textural features. For extraction of single-fibers, (Wu et al., 2003) and (Stein et al., 2008) suggested the fiber tracking method. Bredfeldt et al. realized this approach in the CT-FIRE software (Bredfeldt et al., 2014a). The suggested method is based on identifying nucleation points with subsequent extension to generate fiber branches. Since the fiber tracking algorithm is pixel-wise, it is time-consuming and requires significant computational resources. Our approach is based on skeletonization. To assess morphology of segmented and labeled fibers, we needed to obtain their simplified model. For this aim, we have used a skeletonization algorithm with subsequent pruning of processed fibers to remove artificial spikes and branches. Pruning is also a pixel-wise operation that is time and resource consuming. Our group is planning to continue investigation to increase performance of this fiber tracking method.

Validation of the developed package demonstrated good precision and performance of modules for fiber segmentation, labeling and feature analysis. The results were controlled visually and using other approaches for image processing we used in our previous work (Vasiukov et al., 2020; Senosain et al., 2021). Spatial analysis revealed that tumor cells in indolent and aggressive lung adenocarcinomas are localized in different microenvironments determined by variability of neighboring cells and collagen fibers. The geometrical features of collagen fibers and their spatial distribution play important roles in invasion and metastasis of tumor cells. In addition, co-localization of cancer cells with shorter fibers in aggressive tumors may indicate involvement of these cells in the processes of collagen degradation and ECM remodeling. Moreover, changes in geometrical features of collagen fibers designates modulation of ECM physical properties that are related with increased invasion and metastasis of tumor cells (Provenzano et al., 2009; Frantz et al., 2010; Conklin et al., 2011; Wen et al., 2014; Hanley et al., 2016). Further investigation is needed to decipher this phenomenon.

In summary, automated, robust and efficient methods of computational image analysis allow exploration of complex structural patterns in the tissue related to normal and pathological states. Our approach was designed to elucidate cell-ECM relationships through spatial analysis. Our results show that our developed approach is efficient and can be used to extract of complex structural data related to disease progression and patient outcome.

## Data Availability

The original contributions presented in the study are included in the article/Supplementary Material, further inquiries can be directed to the corresponding author.

## References

[R1] Balsat C, Signolle N, Goffin F, Delbecque K, Plancoulaine B, Sauthier P, (2014). Improved Computer-Assisted Analysis of the Global Lymphatic Network in Human Cervical Tissues. Mod. Pathol 27, 887–898. doi:10.1038/modpathol.2013.195 24309324

[R2] Bankhead P, Loughrey MB, Fernández JA, Dombrowski Y, McArt DG, Dunne PD, (2017). QuPath: Open Source Software for Digital Pathology Image Analysis. Sci. Rep 7, 16878. doi:10.1038/s41598-017-17204-5 29203879 PMC5715110

[R3] Bayan C, Levitt JM, Miller E, Kaplan D, and Georgakoudi I (2009). Fully Automated, Quantitative, Noninvasive Assessment of Collagen Fiber Content and Organization in Thick Collagen Gels. J. Appl. Phys 105, 102042. doi:10.1063/1.3116626 24803683 PMC3987166

[R4] Berg S, Kutra D, Kroeger T, Straehle CN, Kausler BX, Haubold C, (2019). Ilastik: Interactive Machine Learning for (Bio)image Analysis. Nat. Methods 16, 1226–1232. doi:10.1038/s41592-019-0582-9 31570887

[R5] Bhat R, and Bissell MJ (2014). Of Plasticity and Specificity: Dialectics of the Microenvironment and Macroenvironment and the Organ Phenotype. Wires Dev. Biol 3, 147–163. doi:10.1002/wdev.130 24719287

[R6] Bredfeldt JS, Liu Y, Conklin MW, Keely PJ, Mackie TR, and Eliceiri KW (2014). Automated Quantification of Aligned Collagen for Human Breast Carcinoma Prognosis. J. Pathol. Inform 5, 28. doi:10.4103/2153-3539.139707 25250186 PMC4168643

[R7] Bredfeldt JS, Liu Y, Pehlke CA, Conklin MW, Szulczewski JM, Inman DR, (2014). Computational Segmentation of Collagen Fibers from Second-Harmonic Generation Images of Breast Cancer. J. Biomed. Opt 19, 16007. doi:10.1117/1.JBO.19.1.016007 24407500 PMC3886580

[R8] Butcher DT, Alliston T, and Weaver VM (2009). A Tense Situation: Forcing Tumour Progression. Nat. Rev. Cancer 9, 108–122. doi:10.1038/nrc2544 19165226 PMC2649117

[R9] Carpenter AE, Jones TR, Lamprecht MR, Clarke C, Kang IH, Friman O, (2006). CellProfiler: Image Analysis Software for Identifying and Quantifying Cell Phenotypes. Genome Biol 7, R100. doi:10.1186/gb-2006-7-10-r100 17076895 PMC1794559

[R10] Chen DZ, Smid M, and Xu B (2002). Geometric Algorithms for Density-Based Data Clustering. Lect Notes Comput. Sc 2461, 284–296. doi:10.1007/3-540-45749-6_28

[R11] Cho A, Howell VM, and Colvin EK (2015). The Extracellular Matrix in Epithelial Ovarian Cancer - A Piece of a Puzzle. Front. Oncol 5, 245. doi:10.3389/fonc.2015.00245 26579497 PMC4629462

[R12] Comin CH, Xu X, Wang Y, Costa Lda. F., and Yang Z (2014). An Image Processing Approach to Analyze Morphological Features of Microscopic Images of Muscle Fibers. Comput. Med. Imaging Graph 38, 803–814. doi:10.1016/j.compmedimag.2014.07.003 25124286 PMC4955614

[R13] Conklin MW, Eickhoff JC, Riching KM, Pehlke CA, Eliceiri KW, Provenzano PP, (2011). Aligned Collagen Is a Prognostic Signature for Survival in Human Breast Carcinoma. Am. J. Pathol 178, 1221–1232. doi:10.1016/j.ajpath.2010.11.076 21356373 PMC3070581

[R14] Czech E, Aksoy BA, Aksoy P, and Hammerbacher J (2019). Cytokit: a Single-Cell Analysis Toolkit for High Dimensional Fluorescent Microscopy Imaging. BMC Bioinformatics 20, 448. doi:10.1186/s12859-019-3055-3 31477013 PMC6720861

[R15] Dietz C, and Berthold MR (2016). KNIME for Open-Source Bioimage Analysis: A Tutorial. Adv. Anat. Embryol. Cel Biol 219, 179–197. doi:10.1007/978-3-319-28549-8_7 27207367

[R16] Erler JT, and Weaver VM (2009). Three-dimensional Context Regulation of Metastasis. Clin. Exp. Metastasis 26, 35–49. doi:10.1007/s10585-008-9209-8 18814043 PMC2648515

[R17] Feichtenbeiner A, Haas M, Büttner M, Grabenbauer GG, Fietkau R, and Distel LV (2014). Critical Role of Spatial Interaction between CD8^+^ and Foxp3^+^ Cells in Human Gastric Cancer: the Distance Matters. Cancer Immunol. Immunother 63, 111–119. doi:10.1007/s00262-013-1491-x 24170095 PMC11029441

[R18] Frangi AF, Niessen WJ, Vincken KL, and Viergever MA (1998). Multiscale Vessel Enhancement Filtering. Lect Notes Comput. Sc 1496, 130–137. doi:10.1007/bfb0056195

[R19] Frantz C, Stewart KM, and Weaver VM (2010). The Extracellular Matrix at a Glance. J. Cel Sci 123, 4195–4200. doi:10.1242/jcs.023820 PMC2995612 21123617

[R20] Goltsev Y, Samusik N, Kennedy-Darling J, Bhate S, Hale M, Vazquez G, (2018). Deep Profiling of Mouse Splenic Architecture with CODEX Multiplexed Imaging. Cell 174, 968–e15. doi:10.1016/j.cell.2018.07.010 30078711 PMC6086938

[R21] Hanley CJ, Noble F, Ward M, Bullock M, Drifka C, Mellone M, (2016). A Subset of Myofibroblastic Cancer-Associated Fibroblasts Regulate Collagen Fiber Elongation, Which Is Prognostic in Multiple Cancers. Oncotarget 7, 6159–6174. doi:10.18632/oncotarget.6740 26716418 PMC4868747

[R22] Harris CR, Millman KJ, van der Walt SJ, Gommers R, Virtanen P, Cournapeau D, (2020). Array Programming with NumPy. Nature 585, 357–362. doi:10.1038/s41586-020-2649-2 32939066 PMC7759461

[R23] Heindl A, Nawaz S, and Yuan Y (2015). Mapping Spatial Heterogeneity in the Tumor Microenvironment: a new era for Digital Pathology. Lab. Invest 95, 377–384. doi:10.1038/labinvest.2014.155 25599534

[R24] Hemler PF, McCreedy ES, and McAuliffe MJ (2004). Performance Evaluation of Multiscale Vessel Enhancement Filtering. Proc. Spie 5370, 1785–1794. doi:10.1117/12.535675

[R25] Himmel LE, Hackett TA, Moore JL, Adams WR, Thomas G, Novitskaya T, (2018). Beyond the H&E: Advanced Technologies for *In Situ* Tissue Biomarker Imaging. ILAR J 59, 51–65. doi:10.1093/ilar/ily004 30462242 PMC6645175

[R26] Holmes S, Kapelner A, and Lee PP (2009). An Interactive Java Statistical Image Segmentation System: GemIdent. J. Stat. Softw 30. doi:10.18637/jss.v030.i10 PMC3100170 21614138

[R27] Hunter JD (2007). Matplotlib: A 2D Graphics Environment. Comput. Sci. Eng 9, 90–95. doi:10.1109/mcse.2007.55

[R28] Järveläinen H, Sainio A, Koulu M, Wight TN, and Penttinen R (2009). Extracellular Matrix Molecules: Potential Targets in Pharmacotherapy. Pharmacol. Rev 61, 198–223. doi:10.1124/pr.109.001289 19549927 PMC2830117

[R29] Jost AP, and Waters JC (2019). Designing a Rigorous Microscopy experiment: Validating Methods and Avoiding Bias. J. Cel Biol 218, 1452–1466. doi:10.1083/jcb.201812109 PMC6504886 30894402

[R30] Leitinger B, and Hohenester E (2007). Mammalian Collagen Receptors. Matrix Biol 26, 146–155. doi:10.1016/j.matbio.2006.10.007 17141492

[R31] Park M, Yampolsky M, Shlakhter O, VanHorn S, Dygulska B, Kiryankova N, (2013). Vessel Enhancement with Multiscale and Curvilinear Filter Matching for Placenta Images. Placenta 34, A12. doi:10.1016/j.placenta.2013.06.041

[R32] Parra ER (2021). Methods to Determine and Analyze the Cellular Spatial Distribution Extracted from Multiplex Immunofluorescence Data to Understand the Tumor Microenvironment. Front. Mol. Biosci 8, 668340. doi:10.3389/fmolb.2021.668340 34179080 PMC8226163

[R33] Paszek MJ, and Weaver VM (2004). The Tension Mounts: Mechanics Meets Morphogenesis and Malignancy. J. Mammary Gland Biol. Neoplasia 9, 325–342. doi:10.1007/s10911-004-1404-x 15838603

[R34] Paszek MJ, Zahir N, Johnson KR, Lakins JN, Rozenberg GI, Gefen A, (2005). Tensional Homeostasis and the Malignant Phenotype. Cancer Cell 8, 241–254. doi:10.1016/j.ccr.2005.08.010 16169468

[R35] Pickup M, Novitskiy S, and Moses HL (2013). The Roles of TGFβ in the Tumour Microenvironment. Nat. Rev. Cancer 13, 788–799. doi:10.1038/nrc3603 24132110 PMC4025940

[R36] Pijanka JK, Markov PP, Midgett D, Paterson NG, White N, Blain EJ, (2019). Quantification of Collagen Fiber Structure Using Second Harmonic Generation Imaging and Two-Dimensional Discrete Fourier Transform Analysis: Application to the Human Optic Nerve Head. J. Biophotonics 12, e201800376. doi:10.1002/jbio.201800376 30578592 PMC6506269

[R37] Provenzano PP, Inman DR, Eliceiri KW, and Keely PJ (2009). Matrix Density-Induced Mechanoregulation of Breast Cell Phenotype, Signaling and Gene Expression through a FAK-ERK Linkage. Oncogene 28, 4326–4343. doi:10.1038/onc.2009.299 19826415 PMC2795025

[R38] Reis-Sobreiro M, Chen JF, Novitskaya T, You S, Morley S, Steadman K, (2018). Emerin Deregulation Links Nuclear Shape Instability to Metastatic Potential. Cancer Res 78, 6086–6097. doi:10.1158/0008-5472.CAN-18-0608 30154147

[R39] Roeder AH, Cunha A, Burl MC, and Meyerowitz EM (2012). A Computational Image Analysis Glossary for Biologists. Development 139, 3071–3080. doi:10.1242/dev.076414 22872081

[R40] Rubbens MP, Driessen-Mol A, Boerboom RA, Koppert MM, van Assen HC, TerHaar Romeny BM, (2009). Quantification of the Temporal Evolution of Collagen Orientation in Mechanically Conditioned Engineered Cardiovascular Tissues. Ann. Biomed. Eng 37, 1263–1272. doi:10.1007/s10439-009-9698-x 19415496 PMC2690830

[R41] Rueden CT, Schindelin J, Hiner MC, DeZonia BE, Walter AE, Arena ET, (2017). ImageJ2: ImageJ for the Next Generation of Scientific Image Data. BMC Bioinformatics 18, 529. doi:10.1186/s12859-017-1934-z 29187165 PMC5708080

[R42] Schapiro D, Jackson HW, Raghuraman S, Fischer JR, Zanotelli VRT, Schulz D, (2017). histoCAT: Analysis of Cell Phenotypes and Interactions in Multiplex Image Cytometry Data. Nat. Methods 14, 873–876. doi:10.1038/nmeth.4391 28783155 PMC5617107

[R43] Senosain MF, Zou Y, Novitskaya T, Vasiukov G, Balar AB, Rowe DJ, (2021). HLA-DR Cancer Cells Expression Correlates with T Cell Infiltration and Is Enriched in Lung Adenocarcinoma with Indolent Behavior. Sci. Rep 11, 14424. doi:10.1038/s41598-021-93807-3 34257356 PMC8277797

[R44] Shi F, and Yang J (2009). Multiscale Vesselness Based Bilateral Filter for Blood Vessel Enhancement. Electron. Lett 45, 1152–1153. doi:10.1049/el.2009.1192

[R45] Stein AM, Vader DA, Jawerth LM, Weitz DA, and Sander LM (2008). An Algorithm for Extracting the Network Geometry of Three-Dimensional Collagen Gels. J. Microsc 232, 463–475. doi:10.1111/j.1365-2818.2008.02141.x 19094023

[R46] Stringer C, Wang T, Michaelos M, and Pachitariu M (2021). Cellpose: a Generalist Algorithm for Cellular Segmentation. Nat. Methods 18, 100–106. doi:10.1038/s41592-020-01018-x 33318659

[R47] van der Walt S, Schönberger JL, Nunez-Iglesias J, Boulogne F, Warner JD, Yager N, (2014). Scikit-image: Image Processing in Python. Peerj 2, e453. doi:10.7717/peerj.453 25024921 PMC4081273

[R48] Vasiukov G, Novitskaya T, Zijlstra A, Owens P, Ye F, Zhao Z, (2020). Myeloid Cell-Derived TGFβ Signaling Regulates ECM Deposition in Mammary Carcinoma via Adenosine-dependent Mechanisms. Cancer Res 80, 2628–2638. doi:10.1158/0008-5472.CAN-19-3954 32312837 PMC7299805

[R49] Virtanen P, Gommers R, Oliphant TE, Haberland M, Reddy T, Cournapeau D, (2020). SciPy 1.0: Fundamental Algorithms for Scientific Computing in Python. Nat. Methods 17, 261–272. doi:10.1038/s41592-019-0686-2 32015543 PMC7056644

[R50] Wen BL, Brewer MA, Nadiarnykh O, Hocker J, Singh V, Mackie TR, (2014). Texture Analysis Applied to Second Harmonic Generation Image Data for Ovarian Cancer Classification. J. Biomed. Opt 19, 096007. doi:10.1117/1.JBO.19.9.096007 26296156 PMC4161736

[R51] Wu J, Rajwa B, Filmer DL, Hoffmann CM, Yuan B, Chiang C, (2003). Automated Quantification and Reconstruction of Collagen Matrix from 3D Confocal Datasets. J. Microsc 210, 158–165. doi:10.1046/j.1365-2818.2003.01191.x 12753098

